# Preparation of Carbon-Silicon Doping Composite Adsorbent Material for Removal of VOCs

**DOI:** 10.3390/ma12152438

**Published:** 2019-07-31

**Authors:** Zhenwei Han, Shunli Kong, Hong Sui, Xingang Li, Zisheng Zhang

**Affiliations:** 1 School of Chemical Engineering and Technology, Tianjin University, Tianjin 300072, China; 2 National Engineering Research Centre of Distillation Technology, Tianjin 300072, China; 3 Collaborative Innovation Center of Chemical Science and Engineering, Tianjin 300072, China; 4 Department of Chemical and Biological Engineering, University of Ottawa, Ottawa, ON K1N 6N5, Canada

**Keywords:** carbon-silicon composite, activated carbon, VOCs, dynamic adsorption, p-xylene, carboxymethyl cellulose

## Abstract

The adsorption-desorption combined process has been considered as a promising method for the industrial VOCs (volatile organic compounds) treatment. Herein, a carbon-silicon composite adsorbent material has been prepared for the removal of VOCs at lower potential flammable risk. The preparation involves two main steps: Extrusion forming and thermal treatment. The carboxymethyl cellulose and silicate were adopted as binder and fire retardant respectively. The molding and inflaming retarding mechanisms were proposed and discussed. Results show that the newly prepared doping combined material is micro-mesoporous with a specific surface area of 729 m^2^/g. The maximum adsorption capacity of carbon-silicon doping combined material to *p*-xylene is observed to be 292 mg/g. The adsorption is found to be favorable, which is well described by the Yoon-Nelson model and Freundlich isotherm. The combined material is also found to possess reversible adsorption to p-xylene; without sacrificing (<2%) too much adsorption capacity after five adsorption-desorption cycles. The composite materials have an increased ignition temperature of at least 40 °C compared with raw carbon material. These findings suggest that the obtained composite material possesses good adsorption capacity and flame-retardant properties.

## 1. Introduction

The emission of volatile organic compounds (VOCs) has been reported to lead to pollution and health issues for both the environment and human beings [1,2,3]. The removal and recovery of VOCs from off-gases is attracting increasing attention from the industry and society. Many different methods have been proposed and developed to treat the VOCs, including absorption, combustion disposal, catalytic oxidation, and membrane separation [4,5,6]. The fixed-bed adsorption is a promising method [7], which could be used individually or combined together with other methods to fully separate the VOCs from the off-gases, allowing the off-gases to be discharged cleanly [8]. In addition, the adsorption process could recover the VOCs through an adsorption-desorption cyclic process [9,10]. The key of the adsorption-desorption process is the adsorbent, which should possess recyclability. The traditional activated carbons are widely used as adsorbents due to its excellent adsorption capacity and relatively low cost [11,12,13]. However, the applications of activated carbons for adsorbing VOCs sometimes lead to the flammable risk or even explosion (when air or oxygen is involved). 

To solve this safety issue, the flame-retardant carbon materials are proposed to be used in fixed adsorption bed to replace traditional materials. Herein, it is expected that the formed adsorbent doped by silicon helps to improve fire resistance under the premise of adequate adsorption capacity. In this work, a facile two-step method (extrusion forming and thermal treatment) is present to fabricate columnar carbon-silicon composite adsorbent. The prepared composite materials show high adsorption capacity and high ignition point. The possible mechanism was discussed. Then, the dynamic adsorption of p-xylene from gaseous mixture was tested and the results were fitted by theoretical models.

## 2. Materials and Methods 

### 2.1. Materials

The activated carbon powder was provided by Calgon Carbon company (Pittsburgh, PA, USA). The p-xylene (analytical grade) was supplied by Tianjin Yuanli Technology Co. Ltd., Tianjin, China. The sodium carboxymethyl cellulose (CMC) and sodium silicate were purchased at their analytical grade from Tianjin Jiangtian Co. Ltd. (Tianjin, China) and Tianjin Rianlon Bohua pharmaceutical and chemical Co. Ltd. (Tianjin, China) respectively.

### 2.2. Technological Process

**Figure 1:**
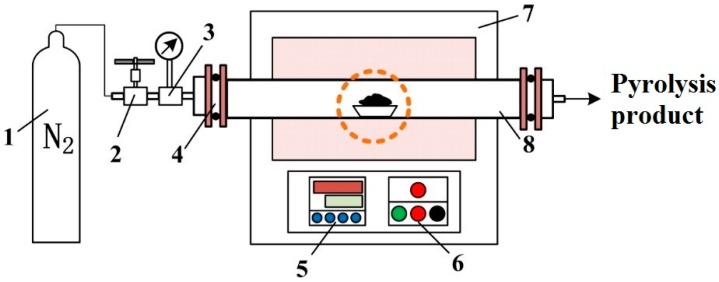
The thermal treatment of molding by tube furnace: (1) nitrogen cylinder, (2) needle valve, (3) gas manometer, (4) sealing flange, (5) control panel, (6) heating switch, (7) thermal-protective coating, (8) quartz tube.

The procedure to produce carbon-silicon composite is given as follows: The binder (CMC) and the raw material (powdered activated carbons, R-AC) were well-blended to increase the contact area. The dissolved silicate was also added to the mixture during this stage. The compression molding of sample was conducted under the pressure of 15 MPa by handy hydraulic pressure (KJ GROUP, Hefei, China). Thermal treatment was proceeded for solidifying bonding agent, which promoted the molding and increased the strength. It was conducted in a tube furnace (OTF-1200X, KJ GROUP, China), as shown in Figure 1. The sample was heated until the temperature reached 200 °C for 60 min in N_2_ atmosphere. We obtained the columned molding composite adsorbents after removing impurities by vacuum drying.

### 2.3. Optimization of Operational Conditions

The molding experiments for composite material were conducted in batch mode to measure the impact of binder and silicon source on the adsorptive property of product. The additive quantity of binder selected in this study was 0.1, 0.2, and 0.3 times of powdered activated carbon at specific intake of silicon source (0.1, 0.3, 0.5 times of raw materials). According to condition, the new carbon–silicon composite adsorption materials are named as AC-Si-x-y, where x is the additive amount of CMC and y is the quality of sodium silicate (the relative quality of raw material). For example, AC-Si-0.2-0.3 stands for the composite adsorption material with addition of 0.2 g CMC and 0.3 g Na_2_SiO_3_ into 1 g activated carbon.

### 2.4. Properties Characterization

The morphologies of raw materials and AC-Si-0.2-0.3 were evaluated by scanning electron microscopy (SEM, Hitachi S-4800, Tokyo, Japan) after dehydration and spray-gold treatment. The morphologies were observed by magnifying the samples 10k times. The chemical characteristics of obtained composite adsorption material were determined by X-ray photoelectron spectroscopy spectra (XPS, ESCALAB 250i, Thermo Fisher Scientific, Waltham, MA, USA). The measured XPS photoelectron spectral was recorded including O1s and Si2p peaks. The surface chemical characteristics of obtained composite adsorption material were determined by infrared spectroscopy Nicolet 6700 (IR, Thermo Nicolet Corporation, Fitchburg, WI, USA). The measured infrared spectroscopy was recorded from 4000 cm^−1^ to 400 cm^−1^.

The adsorption properties of the composite adsorption material were measured by an automatic gas-sorption analyzer (BET, 3H-2000PM2, BeiShiDe Instrument, Beijing, China). The measurement was carried out after a vacuum degassing process at 150 °C for six hours and the standard adsorption-desorption isotherm was measured at −196 °C. The nitrogen-adsorption data was gained at the relative pressure from 0 to 0.994 and the total volume (Vm) of pores was calculated by data at maximum pressure. During analysis of adsorption-desorption data, the Brunauer-Emmett-Teller (BET) model and the density functional theory (DFT) model were used to calculate the specific surface area and the pore diameter distribution, respectively.

### 2.5. Dynamic Adsorption Test

#### 2.5.1. Adsorption and Desorption of p-xylene

**Figure 2:**
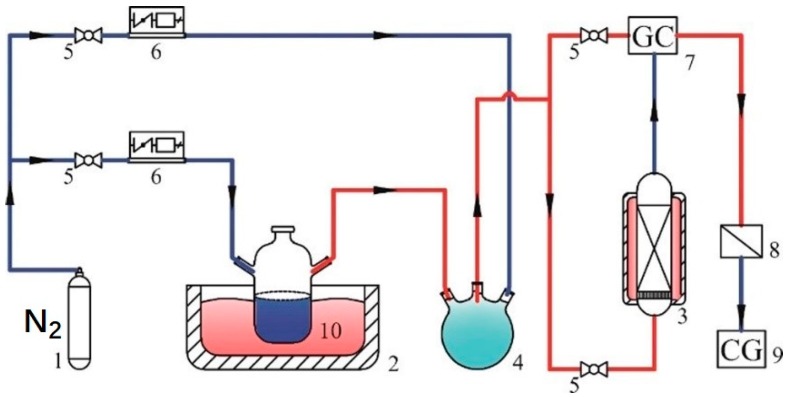
Schematic flowchart of the experimental set up: (1) nitrogen cylinder, (2) thermostat water bath, (3) constant temperature adsorption columns, (4) mix chamber, (5) globe valves, (6) mass flow controllers, (7) gas chromatography, (8) exhaust gas treatment tank, (9) clean gas (10) VOCs mixture generator.

**Figure 3:**
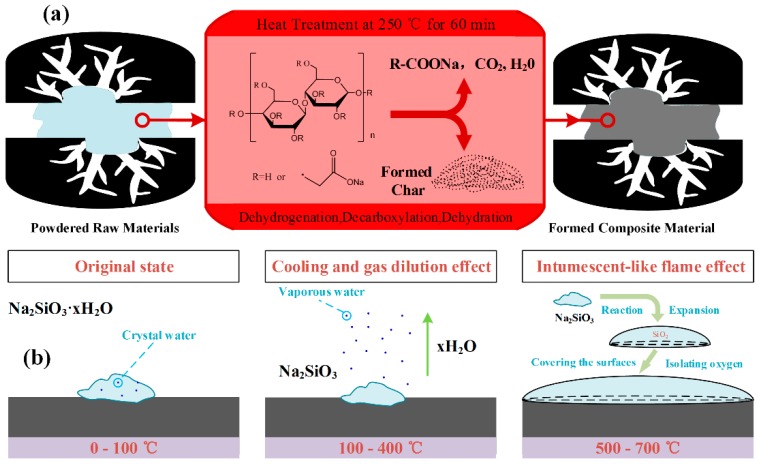
(**a**) Schematic diagram of molding mechanism. (**b**) Schematic diagram of flame-retardant mechanism.

**Figure 4:**
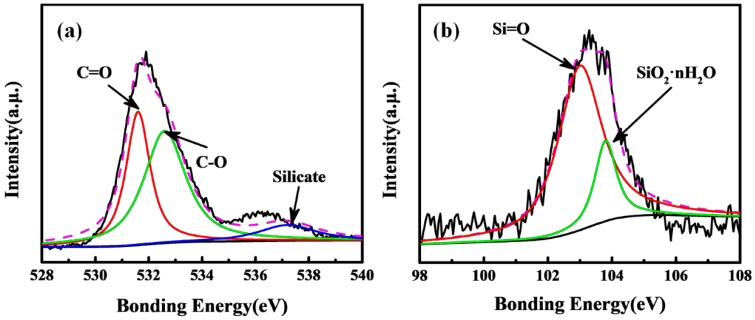
Curve fitting of O1s (**a**) and Si2p (**b**) photoelectron peaks.

**Figure 5:**
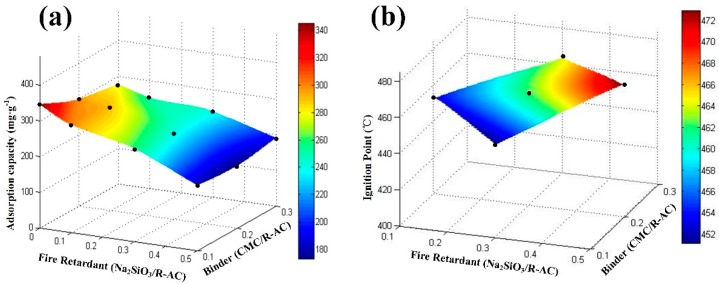
Parameter of optimizing conditions. (**a**) Adsorption capacity of p-xylene on various samples. (**b**) Ignition temperature of representative samples.

**Figure 6:**
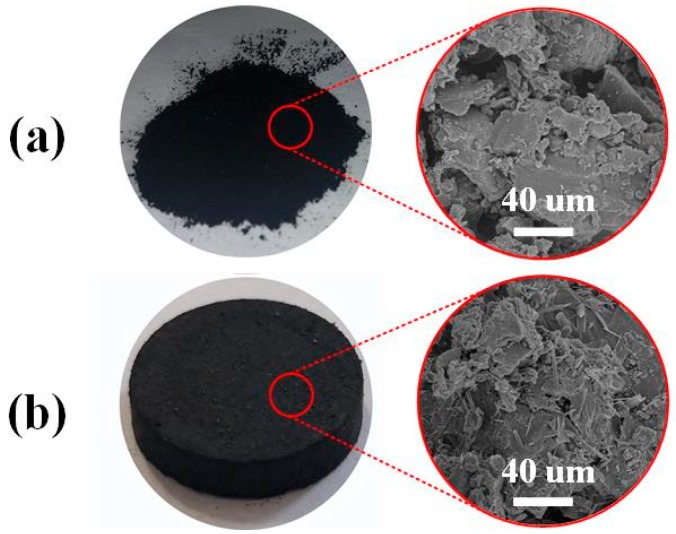
SEM of resultant materials in various stages (**a**) R-AC and (**b**) AC-Si-0.2-0.3.

**Figure 7:**
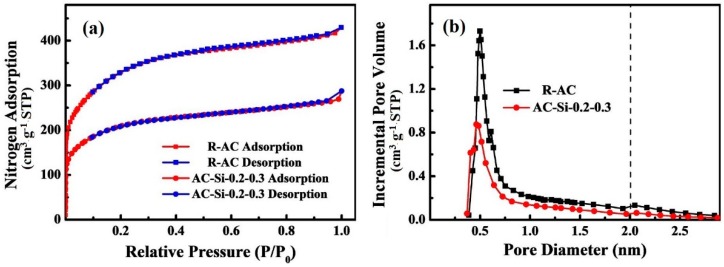
(**a**) The nitrogen adsorption-desorption isotherms and (**b**) the pore diameter distributions of AC-Si-0.2-0.3 and R-AC.

**Figure 8:**
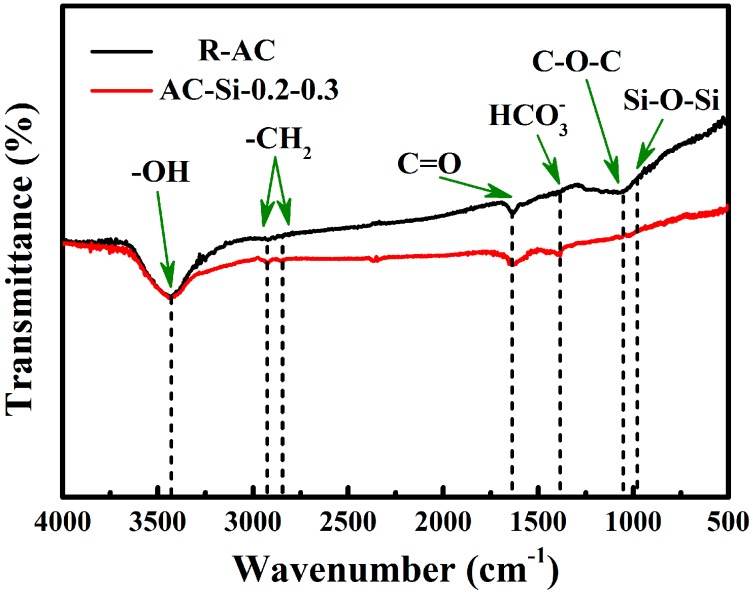
The Infrared Spectroscopy of AC-Si-0.2-0.3 and R-AC.

**Figure 9:**
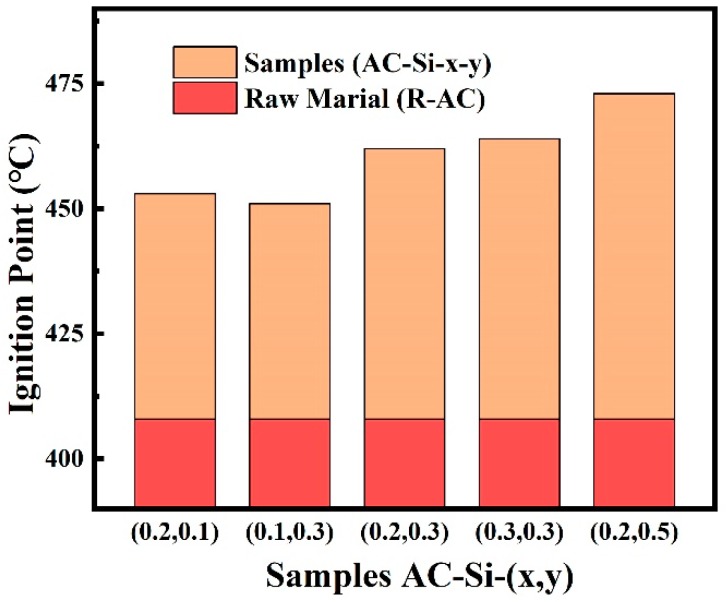
The ignition points of raw material and different samples.

**Figure 10:**
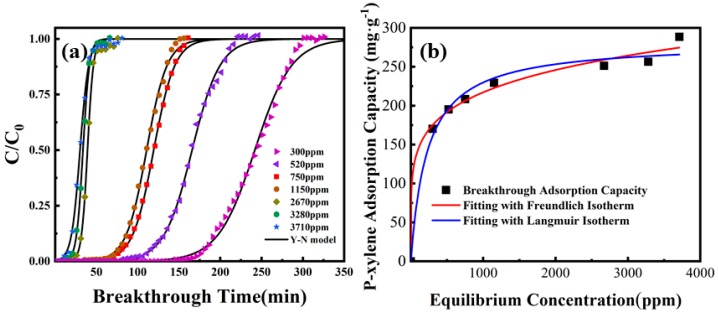
(**a**) Breakthrough curves at different inlet concentration (**b**) Fitted isothermal adsorption curve.

Adsorption capacity of the gained carbon-silicon composite adsorption material was determined by the decrement of p-xylene in gaseous mixture. The dynamic adsorption processes of p-xylene on composite adsorption material were carried out using a custom-made apparatus, shown in Figure 2. The inert carrier gas in this experiment was nitrogen, which was stored in a steel cylinder. A three-way valve was used to split the nitrogen into two streams, both of which were controlled by mass flowmeters (D07-19B, Beijing Sevenstar Electronics Co., Ltd, Beijing, China). One stream flowed through a sealed container (a custom-made apparatus, the shape was shown in Figure 2, Figure 3, Figure 4, Figure 5, Figure 6, Figure 7, Figure 8, Figure 9 and Figure 10) and generated the p-xylene vapor. The other stream was used to dilute the p-xylene vapor for a specified concentration VOCs stream. The concentration of p-xylene stream was measured by a gas chromatography (GC-7900, Shanghai Tianmei Scientific Instrument Co., Ltd, Shanghai, China) through a bypass. The obtained stream containing p-xylene went through the adsorption bed (H:D, 5:1) in usual atmospheric pressure. The saturated adsorption state was considered when the outlet concentration held constant. The uptake of p-xylene by composite adsorption material from gaseous mixture [7,14] at each time, Qt (mg/g), was determined as follows:

(1) $$Q_{t}=\frac{1}{w}\left[F_{A}{\mathrm{MC}}_{0}\left(t-\int_{0}^{t}\frac{C_{t}}{C_{0}}\mathrm{dt}\right)\right],$$

Saturation adsorption capacity, $Q_{\mathrm{ad}}$ (mg/g), was calculated by analogue formula:

(2) $$Q_{\mathrm{ad}}=\frac{1}{w}\left[F_{A}{\mathrm{MC}}_{0}\left(t_{s}-\int_{0}^{t_{s}}\frac{C_{t}}{C_{0}}\mathrm{dt}\right)\right],$$

where w (g) is the weight fixed adsorbent; F_A_ (mmol·min^−1^) is the molar flow rate of influent stream; M (g·mmol^−1^) is the molar mass of p-xylene; t (min) is real-time; C_t_ (ppm) is the effluent concentration at time t; C_0_ (ppm) is the average influent concentration measured before and after an adsorption experiment.

The adsorption process stops when a constant exit concentration was observed. The equilibrium time is influenced by the inlet concentration. The VOCs with different concentrations (from 750 to 3700 ppm) was prepared by changing the bath temperature and flow of inert diluent gas.

The desorption process was conducted when the adsorption was finished. The high-temperature thermal desorption technology was adopted and the sample was heated by tube furnace to 150 °C at flowing pure nitrogen atmosphere. The desorption capacity of p-xylene, $Q_{\mathrm{de}}$ (mg/g), was calculated as follows:

(3) $$Q_{\mathrm{de}}=\frac{M_{\mathrm{ad}}-M_{\mathrm{de}}}{w},$$

The desorption efficiency, $R_{\mathrm{de}}$ (%), was calculated by the following equation:

(4) $$R_{\mathrm{de}}=\frac{M_{\mathrm{ad}}-M_{\mathrm{de}}}{Q_{\mathrm{ad}}w},$$

where $M_{\mathrm{ad}}$ (g) and $M_{\mathrm{de}}$ (g) represent the quality of adsorbent after adsorption and desorption process, respectively. 

To evaluate the desorption properties of AC-Si-0.2-0.3, the adsorption-desorption process was taken up five times.

#### 2.5.2. Adsorption Isotherm

Adsorption equilibrium and adsorption mechanism can be well understood by the adsorption isotherm curves. The Langmuir and Freundlich model are the frequently-used theoretical models for describing the behavior of adsorbate on the composite adsorption material.

Langmuir isotherm model (homogeneous monolayer adsorption) is employed to describe the adsorption process by Equation (5).

(5) $$q_{e}=\frac{q_{m}{\mathrm{bC}}_{e}}{1+{\mathrm{bC}}_{e}},$$

where $q_{e}$ (mg/g) represents the equilibrium absorption capacity, $q_{m}$ (mg/g) the maximum absorption capacity, b Langmuir constant relevant to adsorption free energy, $C_{e}$ (ppm) equilibrium concentration of p-xylene.

The Freundlich isotherm model (non-ideal and reversible adsorption, multilayer heterogeneous adsorption) is employed to describe the adsorption process by Equation (6).

(6) qe=KfCe1n,

where $K_{f}$ represents the coefficient ((mg/g)·(1000/ppm)) being related to the adsorption capacity; n is the Freundlich index (non-dimensional). 

#### 2.5.3. Adsorption Kinetics

Adsorption kinetics of gas or vapors can be predicted by Yoon-Nelson (Y-N) model [15], which is a semi-empirical model [16]. This model can be used to predict the breakthrough curves, shown as follows:

(7) $$-t=\tau +\frac{1}{k_{\mathrm{YN}}}\ln\frac{C\left(t\right)}{C_{0}-C\left(t\right)},$$

where $k_{\mathrm{YN}}$ (min^−1^) is the proportionality constant; τ (min) is the time required 50% adsorbate breakthrough, $C_{0}$ is the influent concentration of p-xylene and C(t) is the effluent concentration.

## 3. Results and Discussion

### 3.1. Silicon Doping on Activated Carbon

Before the doping of silicon onto the activated carbon, the sodium carboxymethyl cellulose (CMC) was added as binder to bind the activated carbon powder together. The silicate was used as silicon source to reduce the ignition point of the carbon adsorbent for adsorbing VOCs gas. This mixture was then heated to allow the binders and silicate to be carbonized into the activated carbons at 250 °C, fabricating the formed composite material, shown in Figure 3a [17]. The flame-retardant mechanism is shown in Figure 3b. The silicate has a small content of hydrated water, which is helpful to improve fire resistance by the cooling effect and gas dilution effect. Especially at lower temperature, this water contributes to the stabilization of adsorbent material. Above all, sodium silicate has an intumescent-like flame retardant effect on combustible material. It not only produces endothermic decomposition reaction, but also has film-forming properties to isolate the air at combustion temperatures of material [18]. 

The characteristics of composite samples were found to be highly dependent on the preparation conditions. According to the adsorption behaviors of *p*-xylene on adsorbent and the increased ignition point, the optimal mixing ratio of powdered carbon, CMC and sodium silicate is determined to be 1:0.2:0.3. Results showed that the binder would make the segmental pores partially blocked, leading to the reduction of the specific area of the newly adsorbent. This pore blocking also reduced the adsorption capacity of the adsorbents. The newly composite materials were further confirmed by high XPS spectra of O1s and Si2p (Figure 4). The silicate corresponds to the O1s peak of 537.5 eV, which did not exist in the raw materials. Moreover, the Si2p peaks were divided into two peaks at 103.0 eV and 103.8 eV, which corresponded to Si=O group and SiO_2_⋅nH_2_O, respectively. Based on the above evidences, it is suggested that the process of doping of silicon is successful and the carbon-silicon composite material has been obtained.

### 3.2. Optimizing Conditions of Preparation

The characteristics of composite samples were found to be highly dependent on the preparation conditions. The adsorption capacity of p-xylene on various doping composite samples and the corresponding kinetic parameters were listed in Table 1. If there is no binder in the preparation process, the material is not suitable for dynamic adsorption of p-xylene because unformed powder activated carbon will plug the pipeline. However, the adsorption capacity amount for the sample without silicate were generally larger. Combining with the adsorption behaviors of p-xylene on this adsorbent, shown in Figure 5a, it is found that increasing the CMC/R-AC ratio from 0.1 to 0.3 exerts a slight effect on the adsorption capacity to p-xylene. However, the addition of silicate significantly reduced adsorption capacity per unit quality. This is the major reason that the imporous silicate has a negative effect and pulls down the mean adsorption capacity.

Figure 5b shows the change of ignition point along with additive quality of silicate, which is the additive fire retardant. It is observed that the doping of silicon into the activated carbon is observed to increase the ignition temperature. The binder (CMC) could not significantly improve ignition point of composite materials and it is noneffective on improving fire retardant. On the contrary, once the addition of silicate increases, the ignition points gradually raise.

Under the premise of strong adsorption ability, the excellent sample possesses higher ignition points and it has advantages in fire resistance. In conclusion, the optimal additions of binder and fire retardant are 20% and 30% of the carbon powder according to the dynamic adsorption capacity and ignition point.

### 3.3. Characteristic of Adsorbing Material

#### 3.3.1. Scanning Electron Microscope (SEM)

Figure 6a,b show the surface morphology of powdered raw material and molding carbon-silicon composite material, respectively. Obviously, both of the two materials have rough surfaces. Although their structures are similar, the obtained composite material has more acicular substance attaching to the irregular surface. This morphology is attributed to the attached binder and mixed silicate.

#### 3.3.2. Nitrogen Adsorption and Desorption (BET)

The nitrogen adsorption-desorption isotherms and pore diameter distribution obtained by calculating in DFT method for the sample were shown in Figure 7. Since the binder plugs part of pores and the imporous silicate pulls down the mean specific surface area, the specific surface area of AC-Si-0.2-0.3 is about 729 m^2^/g, less than the value of R-AC. The type of both isotherms belongs to mixed adsorption type (Type I and Type II isotherm), which indicates the excellent microporosity and some macroporosity. In addition, the hysteresis loops existing in curves were associated with mesoporosity. Most of the pores in the materials were located in the range 1–3 nm with the mean pore sizes are about 1.8 nm. This suggested that the bond forming process and the addition of silicate as fire retardant do not destroy the pore structure of raw material. 

#### 3.3.3. Infrared Spectroscopy (IR)

The infrared spectroscopies of raw material (R-AC) and composite materials (AC-Si-0.2-0.3) were shown in Figure 8. Both the samples possess broad peak at 3444 cm^−1^, which is attributed to the stretching vibrations of hydroxyl groups. The -CH_2_- and C=O exist on the surface, possibly coming from raw materials. The vibration bands assigned to ${\mathrm{HCO}}_{3}^{-}$(as well as ${\mathrm{CO}}_{3}^{2-}$) and Si-O-Si were observed in AC-Si-0.2-0.3. This is different with R-AC and the phenomenon occurred because additive silicate reacts with water and carbon dioxide in the air, so it produced ${\mathrm{HCO}}_{3}^{-}$ and ${\mathrm{CO}}_{3}^{2-}$. Obviously, the Si-O-Si is added to the sample along with sodium silicate.

#### 3.3.4. Ignition Point

The ignition points of raw material and composite materials obtained under different conditions were shown in Figure 9. The doping of silicon into the activated carbon is observed to increase the ignition temperature at least 40 °C. This method of preparing C–Si composite absorbent materials is benefited to improving flame resistance. 

### 3.4. Adsorption Behaviors to p-xylene

#### 3.4.1. Effects of Inlet Concentrations

The dynamic adsorption of p-xylene on this composite adsorbent was shown in Figure 10a. The breakthrough curves (parameters shown in Table 2) were found to be well-matched with the Y-N model (R^2^ > 99%) and it was used to predict the adsorption kinetics of p-xylene upon AC-Si-0.2-0.3. At given feed flow rate, increasing the initial concentration lead to the reduction in time τ and breakthrough time. This suggests that higher p-xylene concentration contributes to mass transfer and accelerate the bed penetration.

Figure 10b depicted the equilibrium absorption capacity of the xylene on composite materials and their nonlinear fitting isotherm results of Langmuir and Freundlich models. The good fitting results by the Freundlich isotherm equation (R^2^ = 99.01%) suggested that this adsorption process of xylene onto heterogeneous surface was multilayer adsorption. 

#### 3.4.2. Cycle Performance

**Figure 11:**
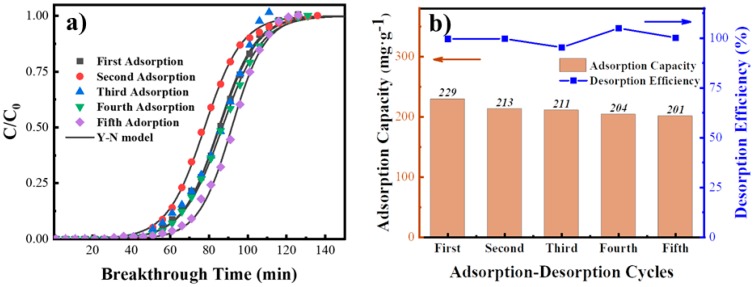
(**a**) Breakthrough curves during the cycle experiment. (**b**) Adsorbing capacity and desorption efficiency.

Figure 11a,b show the breakthrough curves of adsorption-desorption cycles and performance analysis. It is also observed that the initial adsorption capacity was determined to be 229 mg/g. The indistinguishable breakthrough curves indicated that the carbon-silicon composite material has better cycle stability. The slow decline in adsorption capacity also prove that the stability is favorable and the fifth cycle adsorption capacity is about 201 mg/g, which has dropped only 1.5% for the last test. Furthermore, the desorption process of p-xylene has a high desorption efficiency. Almost all of adsorbates are removed by thermal desorption method. The desorption efficiency is closed to 98% and this composite material is appropriate for adsorption-desorption separation process.

## 4. Conclusions

In this work, a molding carbon-silicon composite adsorption material was manufactured by a two-step method. The obtained adsorbent is proved to be a micro-mesoporous material with specific surface area of 729 m^2^/g. The binder (SCMC) contributes to connect different R-AC particles together and form the columnar adsorbent. The silicate acts as fire retardant through increasing ignition point of composite materials. The doping of silicon into the activated carbon is observed to increase the ignition temperature at least 40 °C. This kind of composite adsorbent material was proved to possess the adsorption capacity to p-xylene (288 mg/g at saturation). It is also found that this material performs well in adsorption-desorption cycles (desorption ratio > 98%) for the separating the VOCs from off-gas. This carbon-silicon composite material might be applied to fixed-bed adsorption for VOCs removal and recovery from gaseous mixture. 

## Figures and Tables

**Table 1:** Adsorption capacity for various samples and corresponding kinetic parameters.

Samples	Adsorption Capacity	Y-N Model Fitting Parameters		
	(mg·g^−1^)	K_YN_ (min^−1^)	τ (min)	*R^2^ (%)
AC-Si-0.1-0	345.3	0.073	115.4	99.68
AC-Si-0.2-0	298.6	0.109	67.4	99.83
AC-Si-0.3-0	277.2	0.140	37.3	99.22
AC-Si-0.1-0.1	300.2	0.078	130.7	99.90
AC-Si-0.2-0.1	288.5	0.074	120.5	99.89
AC-Si-0.3-0.1	253.3	0.060	115.6	99.50
AC-Si-0.1-0.3	258.4	0.071	114.1	99.43
AC-Si-0.2-0.3	240.9	0.081	110.0	99.88
AC-Si-0.3-0.3	238.9	0.073	112.2	99.82
AC-Si-0.1-0.5	190.3	0.077	99.1	99.95
AC-Si-0.2-0.5	180.9	0.065	85.5	99.94
AC-Si-0.3-0.5	173.4	0.074	83.0	99.87

* The R^2^ stands for adjusted r-squared.

**Table 2:** Y-N model and isotherms parameters for p-xylene adsorption on AC-Si-0.2-0.3.

Yoon-Nelson Model								Langmuir		Freundlich	
C_0_	300	520	750	1150	2670	3280	3710	-	-	-	-
K_YN_	0.04	0.06	0.08	0.08	0.28	0.25	0.19	qm	293	Kf	68.63
τ	241.7	165.8	119.3	110.7	39.1	33.7	30.1	b	0.00317	n	5.94
*R^2^	99.59	99.80	99.89%	99.81%	99.77%	99.92%	99.80%	R^2^	98.23%	R^2^	99.01%

* The R^2^ stands for adjusted r-squared.
